# Microhabitat selection in the common lizard: implications of biotic interactions, age, sex, local processes, and model transferability among populations

**DOI:** 10.1002/ece3.2138

**Published:** 2016-04-24

**Authors:** Miguel Peñalver‐Alcázar, Pedro Aragón, Merel C. Breedveld, Patrick S. Fitze

**Affiliations:** ^1^ Departamento de Biodiversidad y Biología Evolutiva Museo Nacional de Ciencias Naturales (MNCN, CSIC) José Gutiérrez Abascal 2 28006 Madrid Spain; ^2^ Instituto Pirenaico de Ecología (IPE, CSIC) Avda. Ntra. Señora de la Victoria, s/n 22700 Jaca Spain; ^3^ Departamento de Biogeografía y Cambio Global Museo Nacional de Ciencias Naturales (MNCN‐CSIC) José Gutiérrez Abascal 2 28006 Madrid Spain; ^4^ Department of Ecology and Evolution University of Lausanne Le Biophore 1015 Lausanne Switzerland; ^5^ Fundación Araid, Edificio CEEI Aragón María de Luna 11 50018 Zaragoza Spain

**Keywords:** Abiotic and biotic factors, intraspecific interactions, model transferability, space use models, *Zootoca vivipara*

## Abstract

Modeling species' habitat requirements are crucial to assess impacts of global change, for conservation efforts and to test mechanisms driving species presence. While the influence of abiotic factors has been widely examined, the importance of biotic factors and biotic interactions, and the potential implications of local processes are not well understood. Testing their importance requires additional knowledge and analyses at local habitat scale. Here, we recorded the locations of species presence at the microhabitat scale and measured abiotic and biotic parameters in three different common lizard (*Zootoca vivipara*) populations using a standardized sampling protocol. Thereafter, space use models and cross‐evaluations among populations were run to infer local processes and estimate the importance of biotic parameters, biotic interactions, sex, and age. Biotic parameters explained more variation than abiotic parameters, and intraspecific interactions significantly predicted the spatial distribution. Significant differences among populations in the relationship between abiotic parameters and lizard distribution, and the greater model transferability within populations than between populations are in line with effects predicted by local adaptation and/or phenotypic plasticity. These results underline the importance of including biotic parameters and biotic interactions in space use models at the population level. There were significant differences in space use between sexes, and between adults and yearlings, the latter showing no association with the measured parameters. Consequently, predictive habitat models at the population level taking into account different sexes and age classes are required to understand a specie's ecological requirements and to allow for precise conservation strategies. Our study therefore stresses that future predictive habitat models at the population level and their transferability should take these parameters into account.

## Introduction

Disentangling different abiotic and biotic factors that rule the distribution of species is central to understanding the evolution and ecology of species (Grinnell 1914; Hutchinson 1957; Dunson and Travis 1991; Jablonski 2008) and for their conservation. Predictive habitat models allow identifying suitable areas for species reintroduction or population reinforcement (Engler et al. 2004; Steury and Murray 2004), delimiting priority areas for species conservation (Wilson et al. 2011), predicting extinction risks (Hof et al. 2011), and elucidating the impacts of habitat fragmentation (Santos et al. 2008). In the last decades, species distribution models have been increasingly used and became more sophisticated due to important development in analytical tools, the availability of better environmental and geographic information, and the greater availability of distributional data of species (Guisan and Zimmermann 2000; Guisan and Thuiller 2005). Since these models require precise information, which is generally not available at smaller scales, time‐consuming and expensive to acquire, a bias toward the development of distribution models at large scales exists, at resolution ranging from 10 to 50 km^2^ (Araújo and Guisan 2006; Guisan et al. 2006). However, habitat selection generally occurs at small spatial scales and local conditions may trigger local adaptation and differences due to phenotypic plasticity (Stearns 1989; Kawecki and Ebert 2004). Therefore, local conditions may importantly affect spatial distribution patterns of species (e.g., biotic interactions; Araújo and Luoto 2007; Aragón and Sánchez‐Fernández 2013) and integrating abiotic and biotic factors, including habitat type, and intra‐ and interspecific interactions may crucially improve model accuracy (Pearson and Dawson 2003; Godsoe and Harmon 2012; González‐Salazar et al. 2013).

In this study, we investigated the roles of sex, age, and biotic interactions, and the potential effect of local differences at a small geographic scale in spatial distribution models. Since reptiles are highly susceptible to local abiotic and biotic conditions (Sinervo and Adolph 1989; Lorenzon et al. 1999; Civantos and Forsman 2000), and to the presence of conspecifics (Stamps 1988, 1991), they are especially suited for this study. Therefore, we used the common lizard (*Zootoca vivipara*) as a study species. Common lizards have well‐known life history and exhibit high population densities and a marked age structure (Pilorge et al. 1983; Van Damme et al. 1986; Pilorge 1987; Sorci et al. 1996). Moreover, migration, even among close populations, is rare (Massot et al. 1992). Therefore, it is an ideal species for testing the importance of biotic parameters, biological interactions, and the potential implication of local processes for spatial distribution. We recorded the spatial position of individuals belonging to three classes (adult females, adult males, and yearlings), and abiotic and biotic parameters at the microhabitat scale in three different populations. We performed space use models at a local scale and tested model transferability among populations and the relevance of microhabitat‐related factors (abiotic and biotic parameters), intraspecific interactions, spatial structure, and local differences for predicting spatial distribution. Moreover, we tested for local differences in the importance of these parameters (i.e., significant interactions between populations and predictors) and quantified the amount of explained variance potentially attributable to local adaptation and/or phenotypic plasticity. Our measurement corresponds to the upper limit of variation potentially explained by local processes. In order to avoid problems derived from the presence of spatial autocorrelation, we also accounted for the inherent spatial structure (Lennon 2000; Diniz‐Filho et al. 2003) by considering spatial variability (i.e., spatial filters) in the predictive models (Diniz‐Filho et al. 2003; Borcard et al. 2004; Griffith and Peres‐Neto 2006).

We ran space use models for the three abovementioned lizard classes, which were determined based on body size and coloration. Considered abiotic parameters were soil temperature and soil humidity, and biotic parameters included different measures of vegetation coverage. Intraspecific interactions between age classes, sexes, or both are known to play a key role in space use in lizards (e.g., Stamps 1991; Aragón et al. 2004), and experimental evidence in *Z. vivipara* shows that intra‐ and interage class interactions affect space use (Aragón et al. 2006a, 2006b). Therefore, we took into account intraspecific relationships by including the distributions of the other two lizard classes as surrogates of the sociobiological relationships. To estimate the potential importance of local processes, we analyzed differences among populations in the relationship between biotic and/or abiotic parameters and the lizard distribution. We also evaluated model transferability and we estimated its contribution to the prediction accuracy. In the presence of local processes, such as local adaptation and/or phenotypic plasticity, we predicted: (1) significant differences among populations in the relationship between lizard space use and abiotic, biotic, and/or sociobiological factors; and (2) lower predictive capacity of models when projected to other populations than when projected to a portion of the model population that was randomly excluded from model building. In addition, we quantified the relative importance of abiotic and biotic parameters, and biotic interactions to understand their contribution to the spatial distribution. Finally, we explored differences in space use and local environmental conditions among lizard classes to test the reliability of predictive habitat models using the presence/absence data at microscale.

## Material and Methods

### Study species

**Figure 1:**
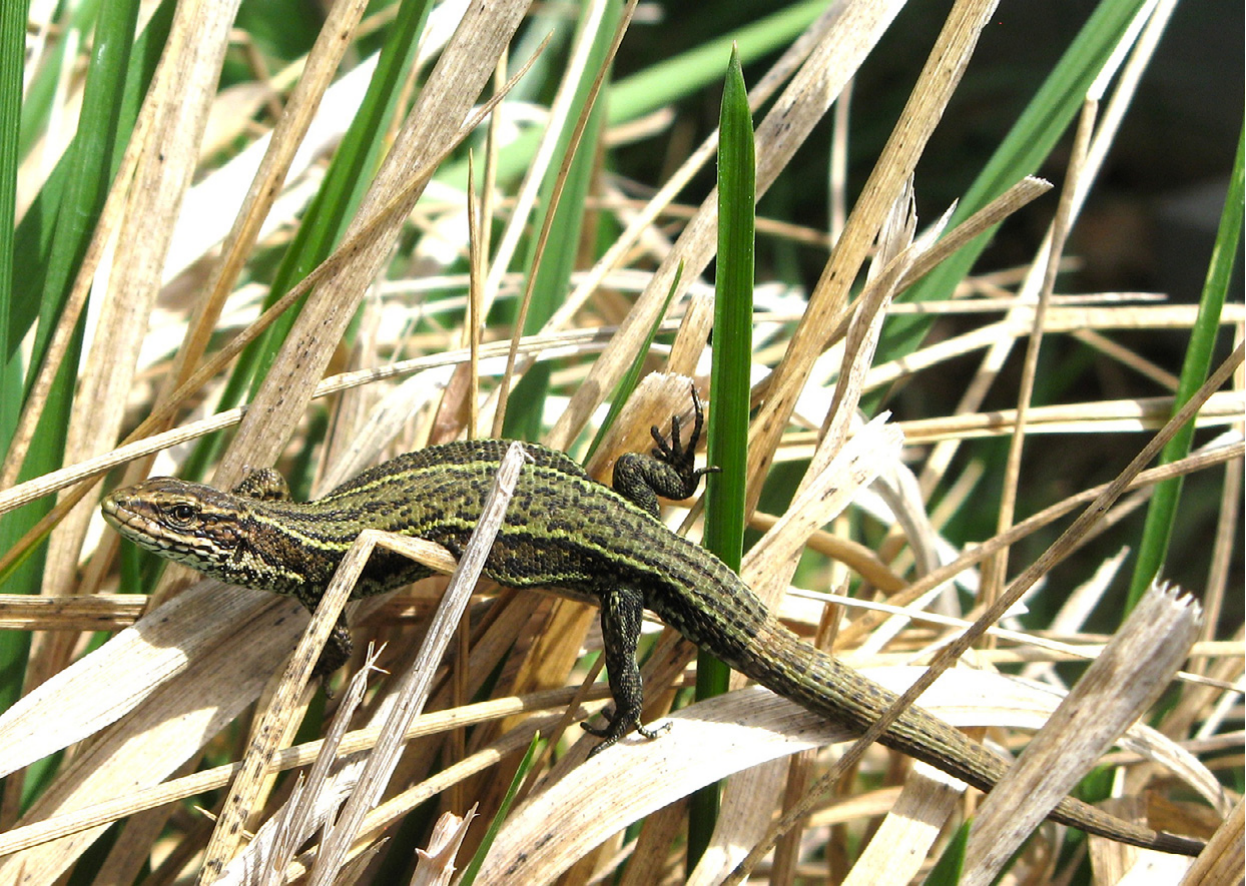
Male common lizard from a population in the central Pyrenees. Photograph: Merel Breedveld.

The common lizard (Fig. 1), *Zootoca vivipara* (Lichtenstein, 1823), is a small ground‐dwelling lacertid lizard (adult snout‐to‐vent length: 45–70 mm). It is widespread across Europe and northern Asia. In the Iberian Peninsula, it is found in the Euro‐Siberian region, and its elevational range is between the sea level and 2400 m a.s.l. (Pleguezuelos et al. 2002). *Z. vivipara* commonly inhabits peat bogs and humid heathlands, as well as places with high moisture substrates such as meadows and grasslands with a predominance of herbs (Pilorge 1987). Three age classes can be distinguished based on body size (Pilorge 1987; Massot et al. 1992) and coloration (Vercken et al. 2007): juveniles, yearlings, and adults. At birth, juveniles are melanic; thereafter, they gradually obtain a pale green‐gray coloration observed in yearlings and reach a typical adult coloration after the second or third hibernation. Females can live up to 10–11 years and live on average 5–6 years, whereas males can live up to 7 years and live on average 3–4 years (Massot et al. 2011). Experimental evidence shows that intraspecific interactions mediate dispersal, space use, and behavior (Léna et al. 1998; Aragón et al. 2006a, 2006b; Cote et al. 2007, 2008; Le Galliard et al. 2008).

### Study populations

**Figure 2:**
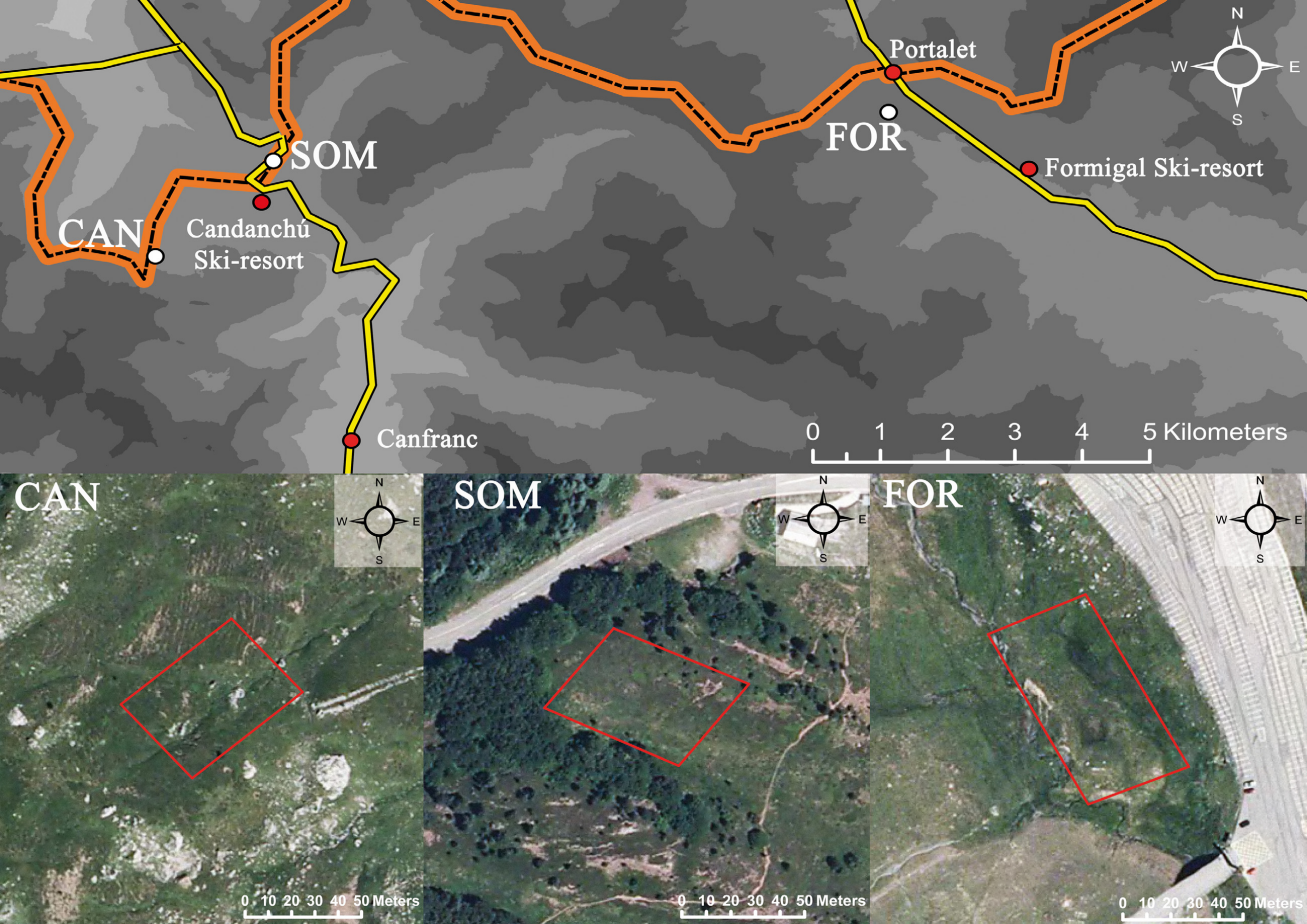
Lidar‐derived 5 m × 5 m digital elevation model (DEM) of the central Pyrenees (Spain) where the populations are located and aerial images of the three study populations of *Z. vivipara* (DEM and images source, PNOA by ^©^ Instituto Geográfico Nacional de España). Perimeters of the study area in the aerial images are delimited in red for the CAN, SOM, and FOR populations. All population images exhibit the same geographic scale and orientation.

We studied three populations (Formigal, Candanchú, and Somport) located in the central Pyrenees (Fig. 2; Huesca, Spain) between 1650 and 1720 m a.s.l. and inhabited by individuals belonging to the same genetic clade (Milá et al. 2013). The Candanchú population (hereafter referred to as CAN; 42°46′51.52″N–0°32′55.35°W, altitude 1670 m a.s.l.) consists of wet heathland that slopes down from southwest to northeast. It is traversed by a small stream, which forms a small flooded area at the northeastern limit of the population. On the southeast, the population is delimited by a rock outcrop and in the northwest by a small hill. Vegetation mainly consists of herbs, and shrubs predominate in the northwestern part. The Somport population (hereafter referred to as SOM; 42°47′41.56″N–0°31′36.18″W, altitude 1650 m a.s.l.) consists of wet heathland and it slopes down north–northwest to east–southeast. It is bordered on the southwest and northwest by a small beech forest and on the northeast by a small rocky outcrop with scattered individuals of mountain pine. Vegetation mainly consists of herbs and shrubs predominate in the northwest. The Formigal population (hereafter referred to as FOR; 42°48′2.96″N–0°24′48.24″W, altitude 1720 m a.s.l.) consists of a bog and wet heathland and slopes down from northwest to southeast. On the southwest, it is bordered by the Gállego River, on the northeast by the slope of an asphalted parking and in the south by the junction of a rill and the Gállego River. Vegetation mainly consists of hydrophilic grasses in the east and herbs in the west.

### Population censuses

We conducted three standardized lizard censuses in 2010 in each population: the first census on the 4th of July in CAN and SOM, and on the 6th of July in FOR; the second census on the 31st of July in FOR and on the 8th of August in CAN and SOM; the third census on the 5th of September in CAN and SOM, and on the 11th of September in FOR. All censuses were carried out between 9:00 and 15:00 GMT + 2, when lizards are most active. In each population, two researchers simultaneously sampled and captured lizards, using a standardized census protocol (see below). The sampled surface covered an area of 2640 m^2^ in CAN and SOM, and 3872 m^2^ in FOR, and sampling precision was 4 × 4 m. The sampling area in FOR was larger since its population nucleus was larger.

Populations were censused with similar sampling effort in each location, by walking horizontally and vertically along transects and in both directions. Lizards were caught by hand and the coordinate was noted. Lizards were brought to the laboratory at the Instituto Pirenaico de Ecología, CSIC (Jaca, Huesca) where body mass (to the nearest 1 mg) and body size (to the nearest 1 mm) were measured, and sex and age class determined. Three age classes were determined based on body size and coloration: adults (i.e., individuals born before 2009), yearlings (i.e., individuals born in 2009), and juveniles (i.e., individuals born in 2010) (see Figure S1). After measurement, lizards were released at the exact capture location. Hatching juveniles exhibit natal dispersal (Clobert et al. 1994), and dispersers and settled juveniles cannot be distinguished unequivocally. Moreover, dispersing individuals can cross unsuitable habitat, and thus including records of dispersers may lead to an overestimation of the suitable habitat, which may compromise the distribution models. Therefore, juveniles were not included in the analyses. The presence of a given lizard class corresponds to at least one lizard belonging to this class and captured in at least one census. The absence of a given lizard class corresponds to no individual of this class captured in the three censuses.

### Sampling of abiotic and biotic parameters

We sampled abiotic and biotic predictors in SOM on July 17th, in FOR on July 18th, and in CAN on July 19th in the middle of each of the 4 × 4 m plots. In order to reduce the effect of rainfall on abiotic parameters, which could potentially result in differences among populations, we sampled populations at least 4 days after the last rain. For each plot, we recorded the percentage covered by rocks, bare soil, herbs, and shrubs (*N *=* *165 at CAN and SOM; *N *=* *242 at FOR; Table S1) since vegetation structure determines the availability of hides and places for thermoregulation in lizards (e.g., Jácome‐Flores et al. 2015; Valenzuela‐Ceballos et al. 2015), including the common lizard (Bauwens and Thoen 1981; Van Damme et al. 1987). We measured proportions following the “point quadrat” method (Henderson 2003). Soil moisture, which is an important factor affecting growth, energetic requirements, behavior, and space use in the common lizard (Grenot et al. 1987; Lorenzon et al. 1999), was measured by drilling out a “soil core” of standard size (10 cm depth). Right after extraction, we stored the soil core in a ziplock plastic bag, to prevent loss of water. In the laboratory, the core's mass was measured, and the bag opened, put into an oven, and dried at 90°C for 4 days. Previous tests showed that the dry weight stabilized after 2 days and did not change from day 3 to day 4 (personal observations). Soil moisture was calculated as the difference between fresh and dry weights and corresponds to water per volume (Table S1). Soil moisture represents all types of moisture (e.g., moisture provided by fog, rain, and hydrologic idiosyncrasies of the locations) and it reflects the hydrologic conditions to which lizards are exposed. We measured soil temperature in the drill hole, right after extraction of the soil core, at a depth of 10 cm using a Fluke 50 thermometer (Fluke Corporation, Everett, Washington) with accuracy ± 0.3°C (Table S1). Temperature was recorded after 30 sec (i.e., after stabilization of the thermometer) and it reflects temperature accumulated by insolation and air temperatures, and thus thermoregulatory conditions of lizards (Huey and Slatkin 1976), a parameter that is crucial for ectotherms.

### Data analysis

#### Degree of dependence between variables

High interdependence between explanatory variables can lead to wrong conclusions (Quinn and Keough 2002). Therefore, we first determined the degree of correlation between predictors using Spearman's rank tests. When two variables were significantly correlated and had *ρ *> 0.9, the variable being more correlated with other predictors was discarded from subsequent analyses (Quinn and Keough 2002).

#### Presence/absence models

We built predictive models of presences/absences using generalized linear models (GLMs) with binomial distribution and logit‐link function (McCullagh and Nelder 1989). GLMs have been recommended when the goal is transferring modeled distributions and predictions in space and time (Araújo and Rahbek 2006; Randin et al. 2006). Space use models based on environmental and sociobiological parameters (hereafter socio‐environmental models) were built for each of the three following lizard classes: adult females (hereafter also referred to as females), adult males (hereafter also referred to as males), and yearlings. The interaction between soil temperature and soil moisture was included because the two parameters may additively or interactively determine lizard space use (Zajitschek et al. 2012). To test whether the space use of a given lizard class is influenced by conspecifics, we included the presence/absence of the other two lizard classes as factors. Models were built either for each study population separately (SOM, CAN, or FOR) or, for model evaluation, for all existing pairs of populations (CAN + SOM, CAN + FOR, or SOM + FOR; see “Model Evaluation” section). To assess the proportion of the variation (deviance in GLMs) exclusively explained by abiotic parameters, by biotic parameters, and the explained variation shared by both, the variation partitioning method proposed by Legendre and Legendre (1998) was used. To test for population differences in relevant parameters, that is, in parameters that significantly predicted the lizard distribution in at least one population, we ran for each lizard class a GLM including data of all populations with population as a factor, the relevant parameters as covariates, and all first‐order interactions between population and the relevant parameters. To confirm differences among populations and lizard classes, we ran a model on the full data set and evaluated the triple interactions between population, lizard class, and the relevant parameters. In these models, we estimated the importance of the effects potentially arising from local processes (i.e., the proportion of variation explained by the significant interactions including population with respect to the total explained variation). Model selection was conducted using backward elimination.

### Model evaluation

We evaluated the accuracy of the predictive models using intrapopulation and interpopulation cross‐evaluation (hereafter evaluation types). For the intrapopulation evaluations, data from each population were randomly split into a training data set consisting of 75% of the presence/absence records and a test data set consisting of the remaining records. Before intrapopulation evaluations, we performed a mixed‐model ANOVA including prevalence as a dependent variable, population as a random factor, data type (test vs. training data) and lizard class as fixed factors and all relevant first‐order interactions to show that prevalence between training and test data was similar. For the interpopulation evaluations, two types of evaluations were performed: First, of each population pair, the joint presence/absence records were used as a training data set and the records of the remaining population (test data) were used for model evaluation. Second, of each population, the presence/absence records were used as a training data set and two independent model evaluations were conducted using the records of the other two populations (test data). Model accuracy was tested using the test data sets and derived from a confusion matrix, that is, a contingency table of the observed and predicted presences and absences (Fielding and Bell 1997). As threshold criteria for the conversion of the continuous probabilities (predictions) into binary predictions (presences/absences), the prevalence (ratio of the number of the presences to the total number of data points in the training data set; Liu et al. 2005), the minimized difference threshold (MDT), the maximized difference threshold (MST; Jiménez‐Valverde and Lobo 2007), and the minimum training presence (MTP; Phillips et al. 2006) criteria were used. Thresholds were calculated using the training data and applied to the test data set used for model evaluation.

Accuracy of the binary predictions was evaluated using four parameters: correct classification rate (CCR; [true positives + true negatives]/*N*), sensitivity (true presence fraction; number of true positives/[number of true positives + number of false negatives]), specificity (true absence fraction; number of true negatives/[number of true negatives + number of false positives]), and area under the receiver operating characteristic (ROC) curve (AUC; Fielding and Bell 1997). The ROC curve is derived by plotting the true‐positive rate (sensitivity) against the false‐positive rate (1, specificity, plotted on the *x*‐axis), across all possible thresholds (Fielding and Bell 1997). AUC has become one of the standard measures to evaluate the accuracy of distribution models. However, this measure cannot be used uncritically and the interpretations derived from this parameter should be accompanied by those derived from their components (sensitivity and specificity; Lobo et al. 2008). AUC values of 1 represent a perfect fit, while 0.5 corresponds to random attribution.

Previous studies showed that accuracy measures (sensitivity, specificity, and CCR) derived with the threshold criteria prevalence, MDT, and MST might be nonindependent (Jiménez‐Valverde and Lobo 2007; Aragón et al. 2010). Therefore, nonindependence was analyzed for all threshold criteria using Pearson correlations, and only noncorrelated threshold criteria (i.e., those not being correlated in any of the conducted model evaluations) were used in the analyses of model transferability.

#### Transferability of distribution models across populations

To evaluate the importance of potentially existing local processes, we tested differences in model transferability among model evaluation types, that is, between intra‐ and interpopulation accuracy measures, using mixed‐model ANOVAs and stepwise backward elimination of nonsignificant terms. Model accuracy measures (sensitivity, specificity, CCR, and AUC) were used as dependent variables; training populations were used as a random factor, and thresholds, lizard class, and evaluation type as fixed factors. All first‐ and second‐order interactions were included in the initial model. Evaluation type consisted of three transferability methods: (1) within population, (2) from two jointly modeled populations toward the third population, and (3) from one population toward each of the other two populations. In the latter case, accuracy measures derived from the same training population were averaged to allow for unbiased comparison with the other evaluation methods. In case of significance of evaluation type, differences among methods were localized using planned post hoc contrasts and Tukey's HSD test (Quinn and Keough 2002).

#### Spatial autocorrelation and variation partitioning

The existence of spatial autocorrelation can alter the results of predictive models by creating false positives, biasing parameter estimates and/or overestimating the contribution of environmental parameters (Lennon 2000; Diniz‐Filho et al. 2003). These potential problems can be avoided by including spatial filters as new parameters in predictive models to explain spatial variation not absorbed by the original parameters (Borcard et al. 2004; Diniz‐Filho and Bini 2005; Griffith and Peres‐Neto 2006). Within this framework, spatial autocorrelation was here taken into account using the specific methodology described by Borcard and Legendre (2002) and Diniz‐Filho and Bini (2005). In brief, a truncated pairwise distance matrix was generated from the population's coordinate system and spatial filters were derived from this matrix using principal coordinate analysis. Spatial filters which exhibited both Moran's *I *> |0.5| and significant Spearman rank correlations with the residuals of the socio‐environmental models were considered relevant (Borcard et al. 2004; Diniz‐Filho and Bini 2005; Griffith and Peres‐Neto 2006). Relevant spatial filters were used to run two model types: a spatial (solely spatial filters as predictors) and a socio‐environmental–spatial model. In both cases, a model was built for each dependent variable (presence/absence records of adult females, adult males, and yearlings) and for each study location separately (CAN, SOM, or FOR). Finally*,* the variation partitioning method was used to assess the proportion of the variation (deviance in GLMs) exclusively explained by the relevant spatial filters, the variation exclusively explained by socio‐environmental parameters, and the explained variation shared by the two factors (i.e., the amount of variation that cannot be exclusively assigned to one parameter or a set of parameters).

Spearman's rank tests, GLMs, mixed‐model ANOVAs, and Pearson correlations were performed using STATISTICA 7.0 (StatSoft 2004). Model evaluations were conducted using the PresenceAbsence package in R (Freeman and Moisen 2008). Spatial autocorrelation analyses and generation of spatial filters were carried out using SAM (Rangel et al. 2010). Model assumptions were tested (normality and homoscedasticity of residuals in ANOVAs and overdispersion in binomial models) and met in all cases.

## Results

### Degree of dependence between measured variables

Shrub coverage was highly correlated with herbaceous coverage, both in CAN and SOM (Spearman test; CAN: *ρ *= −0.923, *P *=* *0.0001; SOM: *ρ *= −0.962, *P *=* *0.0001), and shrubs were therefore not included in the subsequent models. All other correlations exhibited a lower degree of dependence (|*ρ*| for CAN ≤ 0.5, for SOM ≤ 0.4, and for FOR ≤ 0.8), and herbs, bare soil, soil temperature, and soil moisture were thus used in subsequent models.

### Presence/absence models and predictor relevance

Spatial distribution models were run for adult males, adult females, and yearlings. In SOM, a total of 47 adult females, 36 adult males, and 21 yearlings were captured. The prevalence (i.e., the proportion of the sampled plots with the presences) of adult females, adult males, and yearlings was 25.5%, 21.8%, and 12.1%, respectively. In CAN, 14 adult females, 19 adult males, and 7 yearlings were captured, and the prevalence was 7.9%, 11.5%, and 4.2%, respectively. Finally, in FOR, 40 adult females, 40 adult males, and 9 yearlings were captured, and the prevalence was 12.4%, 14.5%, and 3.3%, respectively. The 2010 presence/absence data were positively correlated (Spearman *ρ *= 0.56, *P* < 0.001) with the presence/absence data derived from censuses over 4 years (2007–2010).

In CAN, the presence of adult females was significantly and positively related to herbaceous coverage, and it was significantly and negatively related with soil moisture (Table S2). The presence of adult males was significantly and negatively related with soil temperature and spatial filter no. 12, and positively with spatial filter no. 5. Finally, no parameters significantly predicted the presence of yearlings (Table S2).

In SOM, the presence of adult females was significantly and positively correlated with adult male presence (Table S3). All other parameters were not significantly associated with the presence of adult females. The presence of adult males was significantly and negatively correlated with herbaceous coverage and bare soil, and significantly and positively with adult females presence. No parameters significantly predicted the presence of yearlings.

Finally, in FOR (Table S4), no parameters significantly predicted the presence of adult females. The presence of adult males was positively correlated with soil moisture and negatively with spatial filter no 1. The presence of yearlings was significantly correlated with spatial filter no. 2.

**Figure 3:**
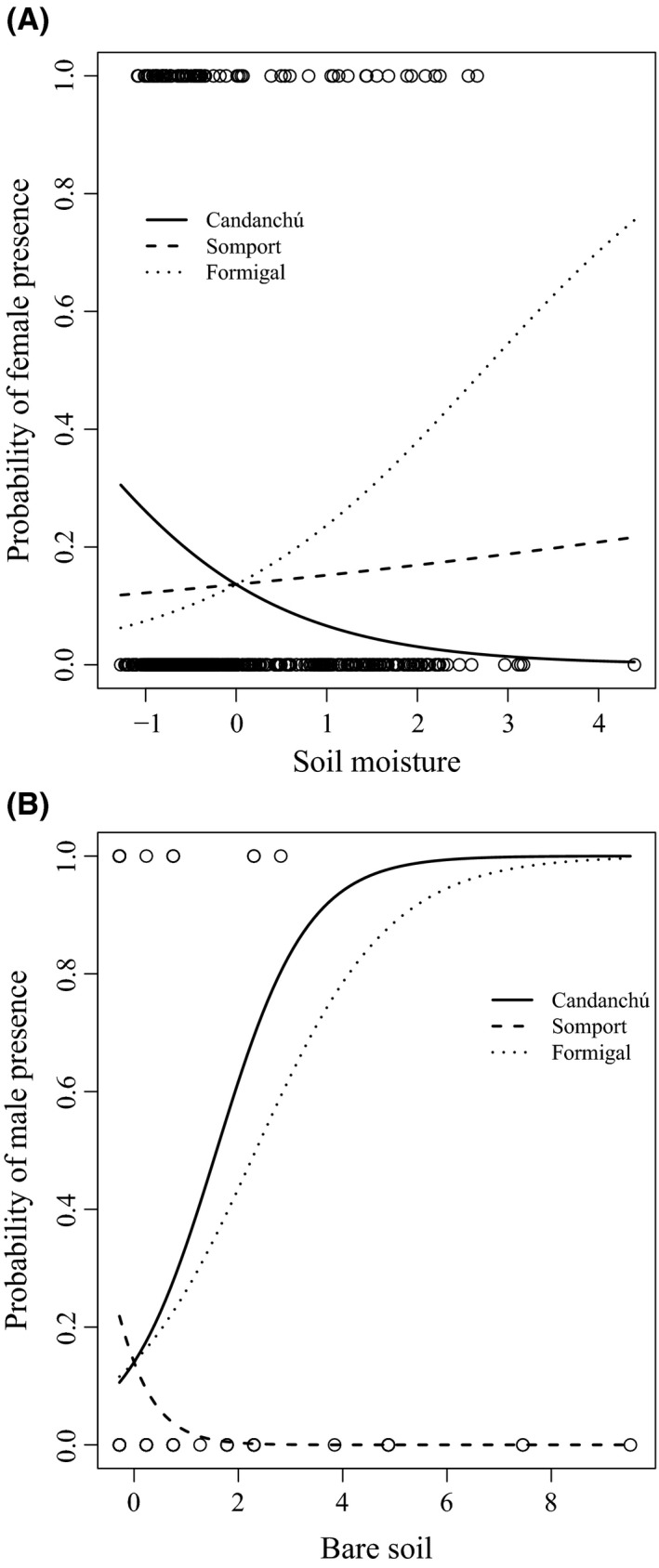
The presence of (A) adult females in relation to soil moisture and (B) adult males in relation to the proportion of bare soil. Given are model predictions for each of the three studied populations derived from the model including data of all three populations. Circles denote observed presences and absences.

The presence/absence models on data of all three populations and including all relevant parameters (i.e., all significant parameters in Tables S2–S4) confirmed that the relationship between the presence of adult females and soil moisture significantly differed between populations (interaction: population × soil moisture χ^2^ = 6.04, df = 2, *P *=* *0.048, 1.26% of the total variation; Fig. 3A). In FOR, the presence of adult females increased with soil moisture, and it decreased in CAN and was unrelated in SOM. The presence of adult females was significantly and positively associated with adult male presence (χ^2^ **=* *6.28, df = 1, *P *=* *0.012, 1.31% of the total variation), and herbaceous coverage, soil temperature, soil moisture, and their interactions with population were not significant (*P *>* *0.05). Pure abiotic parameters explained 2.36%, pure biotic effects 3.41%, and shared effects accounted for 3.50% of the total variation in the presence of adult females.

The presence of adult males was significantly and positively associated with adult female presence (χ^2^ **=* *6.69, df = 1, *P *=* *0.010, 1.34% of the total variation) and there was a significant interaction between population and bare soil (χ^2^ **=* *6.14, df = 2, *P *=* *0.046, 1.17% of the total variation; Fig. 3B). The presence of adult males was positively associated with the proportion of bare soil in CAN and FOR, and it was negatively associated in SOM. All other parameters were not significant (*P *>* *0.05). Pure abiotic parameters explained 1.66%, pure biotic effects 3.88%, and shared effects accounted for 1.06% of the total variation in the presence of adult males. In yearlings, no relevant parameters existed, and thus, the presence/absence models including data of all three populations were not conducted. Models on all populations with adult females and males together confirmed significant differences in the space use among sexes and populations with respect to soil moisture (triple interaction: χ^2^ **=* *6.13, df = 2, *P *=* *0.047) and bare soil (χ^2^ **=* *7.47, df = 2, *P *=* *0.024).

### Model evaluation and transferability

Mixed ANOVAs showed that the proportion of the presences in training and test data did not significantly differ (*F*_1,8_ **=* *2.25, *P *=* *0.272). Moreover, interactions between data type and population (*F*_2,8_ **=* *0.45, *P *=* *0.454) and data type and lizard class (*F*_2,8_ **=* *0.55, *P *=* *0.593) were not significant.

Pearson correlations of accuracy measures obtained with different threshold criteria showed that sensitivity values acquired using the prevalence threshold were significantly correlated with those acquired using the MDT and MST thresholds (*P *<* *0.05, *R *>* *0.96 in both cases). Sensitivity and CCR values obtained with prevalence were correlated with those obtained with MDT thresholds (*P *<* *0.05, *R *>* *0.81 in both cases). No significant correlation was found between values obtained with MTP and those obtained with the other thresholds (*P *>* *0.05, *R *<* *0.42 in all cases). Therefore, for subsequent analyses of model transferability, only accuracy measures (sensitivity, specificity, and CCR) derived with independent thresholds (prevalence, MTP) were used.

**Figure 4:**
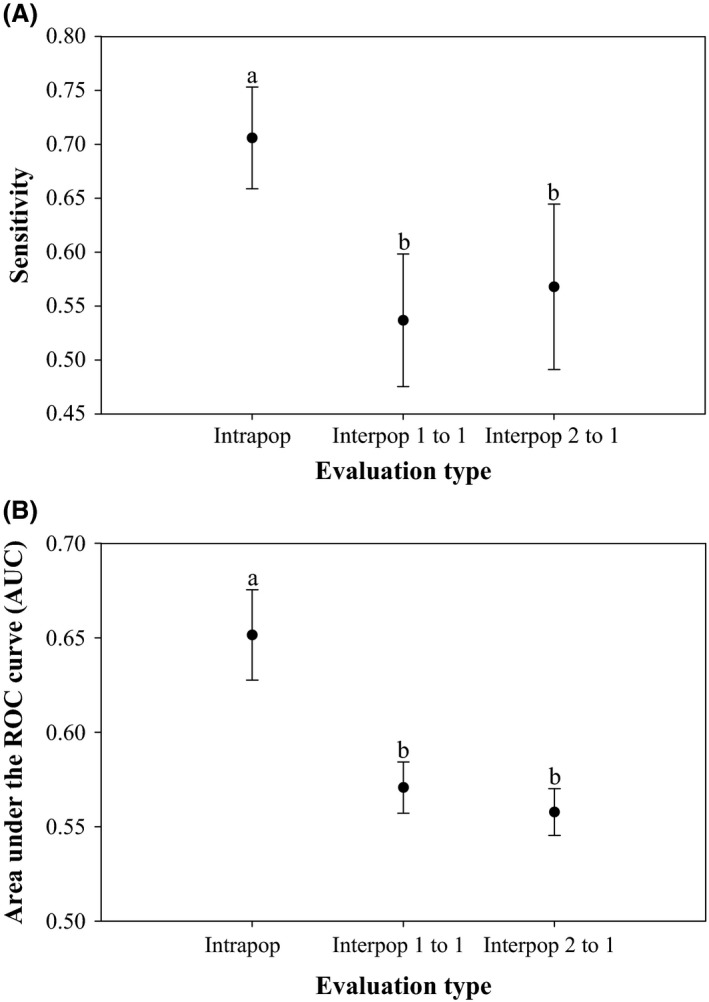
Mean ± SE of (A) sensitivity and (B) area under the ROC curve (AUC) values per evaluation type. Means denoted with different letters indicate significant differences between two evaluation types in planned comparisons. Interpop 1 to 1 denotes models built using data from one population and evaluated in the other populations separately and then averaged. Interpop 2 to 1 denotes models built using data from two joined populations and evaluated in the third population (see “Material and Methods”).

Sensitivity significantly differed among evaluation types (*F*_2,48_ **=* *3.65, *P *=* *0.03) and thresholds (*F*_1,48_ **=* *64.69, *P *<* *0.001), whereas lizard class, population, and all interactions were not significant (*P *>* *0.05 in all cases). Planned comparisons showed that sensitivity was significantly higher in the intrapopulation than in the interpopulation evaluations (*F*_1,48_ **=* *7.07, *P *=* *0.02; Fig. 4A) and no significant differences existed between the interpopulation evaluations (*F*_1,48_ **=* *0.22, *P *=* *0.643; Fig. 4A). Sensitivity was significantly higher when using MTP than when using prevalence as a threshold criterion (mean ± SE; MTP: 0.82 ± 0.04, prevalence: 0.38 ± 0.05).

**Figure 5:**
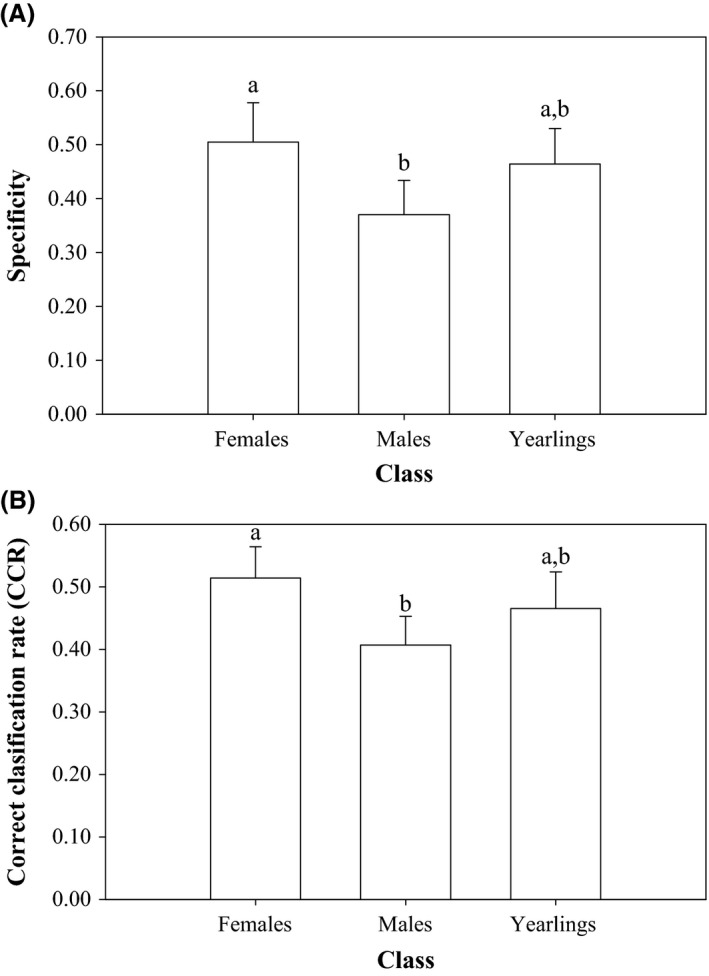
Mean ± SE of (A) specificity and (B) correct classification rate (CCR) per lizard class. Means denoted with different letters indicate significant differences between two lizard classes in post hoc comparisons.

Specificity significantly differed between lizards classes (*F*_2,48_ **=* *4.04, *P *=* *0.024) and thresholds (*F*_1,48_ **=* *145.29, *P *<* *0.001), and type of evaluation did not reach significance (*F*_2,48_ **=* *2.90, *P *=* *0.064). In females, specificity was higher than in males (Fig. 5A), whereas yearlings did not significantly differ from the other two lizard classes (Tukey's HDS: *P *>* *0.05) and specificity was higher when using prevalence than when using MTP (prevalence: mean ± SE* *=* *0.68 ± 0.03, MTP: 0.21 ± 0.03). The population factor and all interactions were not significant (*P *>* *0.05 in all cases).

AUC significantly differed among evaluation types (*F*_2,16_ **=* *4.51, *P *=* *0.028). AUC was significantly higher in the intrapopulation evaluation compared to interpopulation evaluations (planned comparisons: *F*_1,16_ **=* *8.88, *P *<* *0.001; Fig. 4B) and no differences existed between interpopulation evaluations (*F*_1,16_ **=* *0.46, *P *=* *0.708; Fig. 4B). All other factors and their interactions were not significant (*P *>* *0.05 in all cases).

Correct classification rate significantly differed among lizard classes (*F*_2,44_ **=* *4.30, *P *=* *0.020) and thresholds (*F*_1,44_ **=* *41.26, *P *=* *0.023), and there existed a significant interaction between population and threshold (*F*_2,44_ **=* *3.50, *P *=* *0.039). CCR was significantly lower in the three populations when using the MTP instead of the prevalence threshold (Tukey's HDS: *P *<* *0.001), and this effect was more evident in SOM than in CAN (Tukey's HDS: SOM vs. CAN for MTP, *P *<* *0.001). Post hoc comparisons among lizard classes revealed significantly higher CCR in adult females than in adult males (Fig. 5B), whereas CCR in yearlings did not significantly differ from the other two lizard classes (*P *>* *0.05 in both cases; Fig. 5B). Finally, CCR was not significantly affected by evaluation type (*F*_2,44_ **=* *2.81, *P *=* *0.071).

### Spatial autocorrelation, spatial filters, and variation partitioning

Spatial autocorrelation was assessed for each lizard class and population combination. Within combinations, two to five spatial filters were relevant (Table S5), and thus, they were included in the models for each sex and population separately to estimate their relative importance. The proportion of independently explained deviance was greater for socio‐environmental factors than for spatial factors, except in the case of adult males in CAN and FOR (Figure S2), where the proportion explained by spatial factors was higher. In all populations, the proportion of deviance independently explained by the spatial factors was on average 1.9 times greater in males than in females (Figure S2).

## Discussion

To investigate the relevance of biotic interactions and potential implications of local processes in microhabitat selection, we analyzed the relative importance of biotic parameters in space use models and tested for population differences in the contribution of abiotic and biotic factors. The existence of such differences is predicted in the presence of local processes such as local adaptation and/or phenotypic plasticity.

In the space use models of adult females and adult males, the independent contribution of biotic parameters (see Table S1) and biotic interactions explained 1.44 and 2.34 times more variation than the independent contribution of abiotic parameters. The fact that the presence of adults of the opposite sex accounted for 17.5% and 29.0% of the variation explained by the models (in females and males, respectively) suggests that intraspecific interactions were relevant for model building and that biotic interactions may play a role in common lizard habitat selection. The presence of adult conspecifics was significantly and positively associated with the presence of the other sex, especially in SOM (Table S3). Two nonexclusive hypotheses might explain these findings. First, the positive correlation might be the result of individuals selecting the same type of microhabitat independently of the presence of conspecifics, implying that the presence of conspecifics acted as a surrogate predictor of other, here not measured, habitat characteristics. Potential candidate characteristics are food availability, the presence of predators, and wind conditions. Second, “social attraction” can act independently of habitat quality (Stamps 1988, 1991), and a positive correlation can arise due to mutual attraction among conspecifics. Other biotic parameters were of reduced relevance (e.g., herbaceous cover affected presences in one population, but there was no significant overall effect, Tables S2–S4). In general, the biotic parameters and intraspecific interactions that predicted the presence of males and females accounted for more variation than the relevant abiotic parameters, highlighting their importance. Besides the here considered parameters, additional biotic parameters and intraspecific interactions might be relevant, potentially leading to an increase in the variation explained by space use models. For instance, interspecific interactions (e.g., competition and predation) may shape the spatial distribution of organisms (Brown et al. 1996; Svenning et al. 2014). In many populations, *Z. vivipara* lives in sympatry with other reptile species, including competitors (e.g., *Podarcis muralis*) and predators (e.g., snakes). Both may affect the common lizard's behavior, their presence (e.g., they avoid locations with chemical cues from snakes and vipers; Van Damme et al. 1995), and thus their spatial distribution (Mole 2010). Recent studies indicate that including the abundance of predators and/or competitors can improve model accuracy and transferability (Wang and Jackson 2014; Lois et al. 2015). For this reason, we also monitored other reptile species, and their abundance was very low, preventing us from including them in the modes. In fact, the presence of *P. muralis* was detected only twice in FOR, and no other reptile species were detected throughout the study period. Thus, it is unlikely that interspecific interactions with other reptiles determined the observed spatial distribution. Vegetation structure can play an important role for many animal species, given that vegetation provides hides and specific thermoregulatory conditions. In CAN, but not in the other populations, the presence of females was significantly and positively related with the proportion of herbaceous cover. In SOM, the presence of males was significantly and negatively related with the proportion of bare soil (Fig. 3B), indicating that males avoid spots without vegetation, while in FOR and CAN, it was positively but significantly related (Fig. 3B). These interpopulation differences indicate that the relevance of vegetation structure might depend on small‐scale/local idiosyncrasies such as predator abundance, predator type, and abundance of thermoregulatory conditions. For instance, while in some populations vegetation types may provide hides from avian predators (e.g., in CAN from raptors), in other populations it may be used by terrestrial predators (e.g., snake species; Martín and López 1995; Amo et al. 2004), and thus, no preferences for vegetation coverage may exist.

The significant differences among populations in predictor importance and the differences in the predictability among intra‐ and interpopulation evaluations are consistent with the predictions derived from local processes, that is, that individuals of different populations exhibit differences in preferences and behavior. Local processes (estimated by the interactions between population and predictors) accounted for 1.26% of the total variation (population × soil moisture) in adult females and 1.17% of total variation (population × bare soil) in adult males. Accordingly, sensitivity and AUC had higher values in intra‐ than in interpopulation evaluations. Intrapopulation evaluations were above random attribution, whereas interpopulation evaluations were not much better than random attribution (AUC values were close to 0.5). Moreover, the interactions of evaluation type with threshold criteria and lizard class were not significant, showing that the evaluation type effects were robust. Our results therefore indicate that local conditions are responsible for differences in parameter relevance and predictive power. This result is in line with the hypothesis that local processes may affect the predictive power of distribution models (Stockwell and Peterson 2002; Brotons et al. 2004) and with previous findings showing that phenotypic plasticity and local adaptation importantly affect the life history of *Z. vivipara* (Sorci et al. 1996; Lorenzon et al. 1999). The magnitude of effects potentially resulting from local processes (i.e., the interactions between population and soil moisture or bare soil in females and males, respectively) accounted for one‐fifth to one‐fourth of the variation explained by the model (1.26% and 1.17% of the total variation, or 16.8% and 25.16% of the variation explained by the model), and their effect on the spatial distribution was around 1.3% (proportion of total variation in spatial distribution) in males and females. These estimates correspond to the upper limit of effects potentially caused by local adaptation and/or phenotypic plasticity in the studied parameters.

There were significant differences in the space use among lizard classes. In yearlings, the space use was not related with any of the measured parameters, which contrasts to adults. These differences suggest that yearlings may use a larger range of microhabitat, that they may show less precise preferences than adults, or that they may exhibit erratic behavior. Another putative explanation is that some yearlings are still in the dispersal phase, since some individuals do not finish dispersal within the year following their birth (Clobert et al. 1994). Other studies with *Z. vivipara* suggest that yearlings might avoid competition with adults, which can display aggressive behavior toward yearlings (Lecomte et al. 1994; Léna et al. 1998) and could pose a potential risk of cannibalism as observed in other lacertids (e.g., Grano et al. 2011). However, this hypothesis is not supported by our data, given that the presence of adults did not negatively influence the presence of yearlings. Distribution models of adult males showed significantly lower prediction capacity than models of adult females (Fig. 5). These differences may be due to sex‐specific responses to biotic and abiotic factors within localities. Male and female common lizards potentially use habitats in different ways in order to acquire water and heat, as a result of differing resource requirements (Patterson and Davies 1978; Grenot et al. 1987). Overall, our results show that differences among males and females in microhabitat use are of considerable importance and that taking the age and sex of animals into account will improve model reliability. This is especially important when testing species requirements at the population level, given that different habitat use among age classes and sexes could require different conservation strategies. In this context, knowledge about this differential space use may allow to specifically favor a given sex, for example, to rebalance biased sex ratios and to avoid their detrimental effects on population dynamics (Milner‐Gulland et al. 2003; Le Galliard et al. 2005). Also, testing for differences in habitat requirements among age classes may help to design better reintroduction programs. For instance, our results show that colonization success at early stages of the ontogeny may be higher due to less pronounced habitat preferences. Finally, another relevant aspect of our models is the low rate of false positives. This circumstance is of great importance for conservation efforts, where it is crucial not to overestimate organism's presences (Loiselle et al. 2003; Lobo et al. 2008).

Variation partitioning into independent socio‐environmental, spatial, and shared components showed that in most “population × lizard class” combinations, the variation exclusively explained by socio‐environmental parameters was higher than by relevant spatial filters (Figure S2). In fact, spatial filters explained on average only 5.46% (range: 1.12–11.59%) of the total variation. This indicates that the use of space by adult lizards was reasonably well explained by the socio‐environmental models. Still, the significance of certain spatial filters suggests that other environmental variables not considered in this study may play a significant role in the species' space use.

Model accuracy depended on the used threshold and accuracy parameter. The threshold method affected the accuracy estimated using sensitivity, specificity, and CCR. Sensitivity was significantly higher when using MTP and specificity was significantly higher when using prevalence, which is in line with the definition of sensitivity and specificity and their interdependence. The significant interaction between threshold and population on CCR further indicates that accuracy depends on the local characteristics, and thus, determination of an optimal accuracy parameter may be hindered. Consequently, different thresholds and accuracy parameters should be used simultaneously (Hernández et al. 2006).

In summary, our results show that biotic parameters are of great importance and that they can explain more variation than the frequently used abiotic parameters. Moreover, sociobiological relationships (intraspecific interactions) significantly affected the spatial distribution indicating that not taking biological interactions into account may lead to imprecise models and lower prediction capacities. The upper limit of variation potentially explained by local processes reflected a considerable part of the statistical model. Moreover, the significant differences in spatial distribution among age classes indicate that running independent models for different classes of individuals will lead to a better understanding of habitat or space use. Our study indeed shows that age class, sex, biotic parameters, and biological interactions significantly affected the distribution of the individuals at the microhabitat scale and such data should thus be collected and incorporated in future models. Their inclusion may allow to fine‐tune conservation measures.

## Data accessibility

Data available from the Dryad Digital Repository: http://dx.doi.org/10.5061/dryad.4nb46

## Conflict of Interest

None declared.

## Supporting information


**Table S1.** Descriptive statistics of the three study population.
**Table S2–S4.** GLM results.
**Table S5**. Spatial autocorrelation and spatial filters.
**Figure S1.** Sex‐age class body size distribution.
**Figure S2.** Variation partitioning.
