# Supplementary material for: Microhabitat selection in the common lizard: implications of biotic interactions, age, sex, local processes, and model transferability among populations
Source: Ecol Evol. 2016 Apr 24;6(11):3594–607. doi: 10.1002/ece3.2138 (PMC4848056; doi:10.1002/ece3.2138)
Supplement: Supplementary file 1 — Table S1. Descriptive statistics of the three study population. Table S2–S4. GLM results. Table S5. Spatial autocorrelation and spatial filters. Figure S1. Sex‐age class body size distribution. Figure S2. Variation partitioning. [file ECE3-6-3594-s001.doc]

**Supporting information**

**
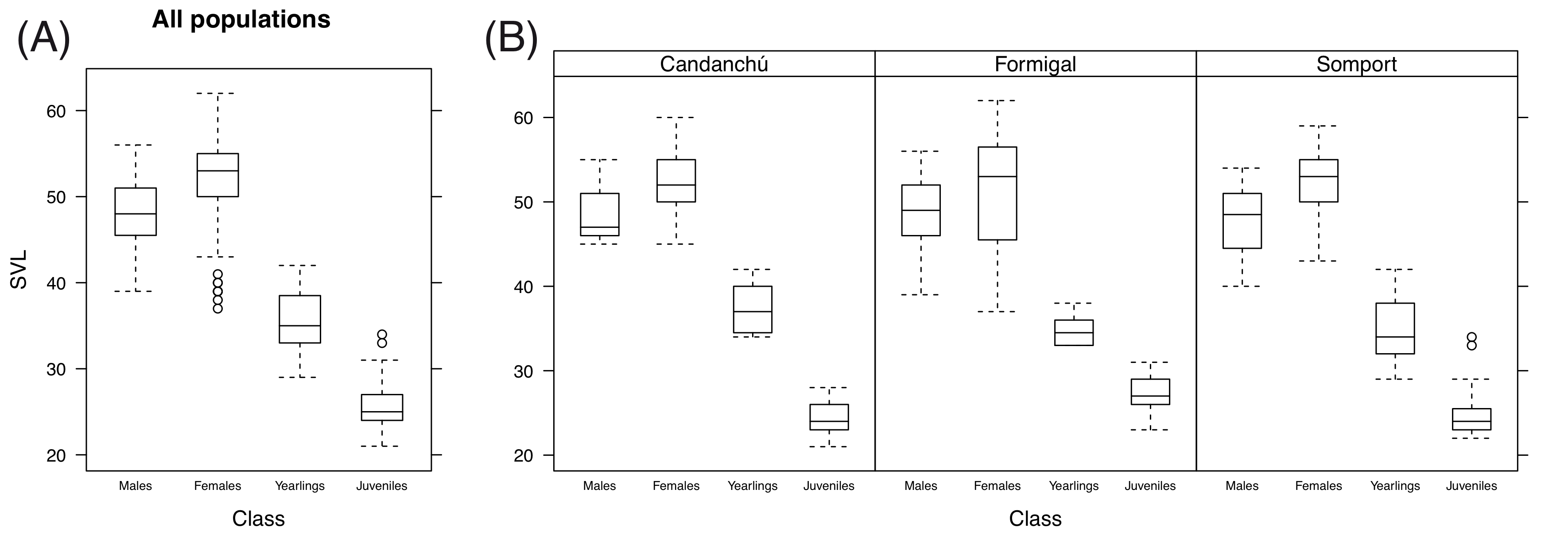
**

Figure S1: Sex-age class body size distribution for (a) all populations and for (b) each studied population and for adult males, adult females, Yearlings and juveniles. Shown are quantile plots and outliers.

Table S1. Descriptive statistics of abiotic and biotic parameters of the three study populations. The minimum, the first quartile (Q_25_), the median, the mean, the third quartile (Q_75_) and the maximum value for each parameter and population are reported. The number of sampling plots for Candanchú and Somport was *N* = 165 and for Formigal *N* = 242.

| Variable | Abiotic | | | |  | Biotic |
| --- | --- | --- | --- | --- | --- | --- |
|  | Rocks (%) | Bare soil (%) | Soil temperature (ºC) | Soil moisture (w/v) |  | Herbaceous (%) |
|  |  |  |  |  |  |  |
| **a. Candanchú** |  |  |  |  |  |  |
|  |  |  |  |  |  |  |
| Min | 0 | 0 | 13.2 | 13.4 |  | 0 |
| Q_25_ | 0 | 0 | 15.0 | 35.9 |  | 50 |
| Median | 0 | 0 | 15.8 | 47.8 |  | 95 |
| Mean | 0.15 | 1.82 | 15.8 | 58.8 |  | 70.36 |
| Q_75_ | 0 | 0 | 16.6 | 69.6 |  | 100 |
| Max | 20 | 50 | 19.2 | 167.2 |  | 100 |
|  |  |  |  |  |  |  |
|  |  |  |  |  |  |  |
| **b. Somport** |  |  |  |  |  |  |
|  |  |  |  |  |  |  |
| Min | 0 | 0 | 12.1 | 6.3 |  | 0 |
| Q_25_ | 0 | 0 | 14.9 | 23.5 |  | 0 |
| Median | 0 | 0 | 15.8 | 30.0 |  | 20 |
| Mean | 0.03 | 1.64 | 16.0 | 32.1 |  | 36.27 |
| Q_75_ | 0 | 0 | 16.8 | 39.7 |  | 75 |
| Max | 5 | 75 | 21.9 | 90.1 |  | 100 |
|  |  |  |  |  |  |  |
|  |  |  |  |  |  |  |
| **c. Formigal** |  |  |  |  |  |  |
|  |  |  |  |  |  |  |
| Min | 0 | 0 | 12.7 | 9.7 |  | 5 |
| Q_25_ | 0 | 0 | 15.1 | 38.7 |  | 95 |
| Median | 0 | 0 | 16.5 | 87.3 |  | 100 |
| Mean | 0.14 | 4.07 | 16.7 | 87.4 |  | 94.24 |
| Q_75_ | 0 | 0 | 18.1 | 128.8 |  | 100 |
| Max | 10 | 95 | 23.5 | 259.4 |  | 100 |
|  |  |  |  |  |  |  |

Table S2. GLM results of the socio-environmental-spatial presence/absence models for adult females, adult males, and yearlings in Candanchú. Test statistics, derived from a likelihood-ratio test, and the standardized regression coefficient (*β* ± standard error), derived from the GLM are shown. Significant parameters are denoted in bold.

| *Variables* | χ^2^ *_1,152_* | *P* | β ± SE |
| --- | --- | --- | --- |
|  |  |  |  |
| ***a. Females*** |  |  |  |
| Intercept | 19.740 | <.0001 | -3.243 ± 0.965 |
| Bare soil | 0.039 | 0.843 | -0.078 ± 0.414 |
| **Herbaceous** | **4.805** | **0.028** | **0.801 ± 0.396** |
| Soil temperature | 2.632 | 0.105 | -0.662 ± 0.411 |
| **Soil moisture** | **9.016** | **0.003** | **-1.623 ± 0.759** |
| Soil temperature x Soil moisture | 0.187 | 0.666 | -0.235 ± 0.522 |
| Spatial filter no 5 | 3.142 | 0.076 | 7.566 ± 4.418 |
| Spatial filter no 8 | 0.411 | 0.521 | -2.795 ± 4.380 |
| Spatial filter no 10 | 0.075 | 0.784 | -1.176 ± 4.279 |
| Spatial filter no 13 | 0.838 | 0.360 | -4.181 ± 4.634 |
| Spatial filter no 31 | 2.848 | 0.092 | -7.085 ± 4.338 |
| Males P/A | 0.649 | 0.420 | -0.425 ± 0.572 |
| Yearlings P/A | 0.202 | 0.653 | 0.321 ± 0.688 |
|  |  |  |  |
| ***b. Males*** |  |  |  |
| Intercept | 18.764 | <.0001 | -2.608 ± 0.770 |
| Bare soil | 3.314 | 0.069 | 0.464 ± 0.227 |
| Herbaceous | 1.598 | 0.206 | 0.475 ± 0.385 |
| **Soil temperature** | **5.034** | **0.025** | **-0.793 ± 0.363** |
| Soil moisture | 0.962 | 0.327 | -0.296 ± 0.316 |
| Soil temperature x Soil moisture | 0.017 | 0.897 | 0.032 ± 0.243 |
| Spatial filter no 2 | 0.001 | 0.977 | -0.134 ± 4.630 |
| Spatial filter no 3 | 0.728 | 0.394 | -3.274 ± 3.879 |
| **Spatial filter no 5** | **6.634** | **0.010** | **9.269 ± 3.764** |
| Spatial filter no 11 | 1.028 | 0.311 | 3.267 ± 3.259 |
| **Spatial filter no 12** | **5.839** | **0.016** | **-9.886 ± 4.336** |
| Females P/A | 0.180 | 0.672 | -0.231 ± 0.571 |
| Yearlings P/A | 0.007 | 0.936 | 0.048 ± 0.583 |

Table S2. Continued

| *Variables* | χ^2^ *_1,154_* | *P* | β ± SE |
| --- | --- | --- | --- |
|  |  |  |  |
| ***c. Yearlings*** |  |  |  |
| Intercept | 30.500 | <.0001 | -2.894 ± 0.746 |
| Bare soil | 0.189 | 0.664 | 0.196 ± 0.404 |
| Herbaceous | 0.083 | 0.774 | -0.176 ± 0.610 |
| Soil temperature | 0.992 | 0.319 | -0.540 ± 0.551 |
| Soil moisture | 1.295 | 0.255 | 0.572 ± 0.488 |
| Soil temperature x Soil moisture | 0.013 | 0.908 | -0.037 ± 0.319 |
| Spatial filter no 3 | 0.043 | 0.835 | -1.233 ± 5.952 |
| Spatial filter no 4 | 0.626 | 0.429 | 5.007 ± 6.442 |
| Spatial filter no 9 | 0.048 | 0.826 | 1.237 ± 5.642 |
| Females P/A | 0.784 | 0.376 | 0.597 ± 0.616 |
| Males P/A | 0.001 | 0.973 | 0.020 ± 0.570 |
|  |  |  |  |

Table S3. GLM results for the socio-environmental-spatial presence/absence models for adult females, adult males, and yearlings in Somport. Test statistics, derived from a likelihood-ratio test, and the standardized regression coefficient (*β* ± standard error), derived from the GLM are shown. Significant parameters are denoted in bold.

| *Variables* | χ^2^ *_1,155_* | *P* | β ± SE |
| --- | --- | --- | --- |
|  |  |  |  |
| ***a. Females*** |  |  |  |
| Intercept | 3.074 | 0.080 | -0.488 ± 0.277 |
| Bare soil | 0.315 | 0.575 | 0.122 ± 0.197 |
| Herbaceous | 2.116 | 0.146 | 0.308 ± 0.213 |
| Soil temperature | 2.870 | 0.090 | -0.382 ± 0.231 |
| Soil moisture | 1.068 | 0.301 | -0.234 ± 0.231 |
| Soil temperature x Soil moisture | 0.055 | 0.814 | 0.05 ± 0.200 |
| Spatial filter no 6 | 3.546 | 0.060 | 4.662 ± 2.515 |
| Spatial filter no 27 | 1.419 | 0.234 | -3.009 ± 2.551 |
| **Males P/A** | **6.733** | **0.010** | **0.572 ± 0.219** |
| Yearlings P/A | 3.526 | 0.060 | 0.508 ± 0.267 |
|  |  |  |  |
| ***b. Males*** |  |  |  |
| Intercept | 16.612 | <.0001 | -1.460 ± 0.444 |
| **Bare soil** | **8.281** | **0.004** | **-2.915 ± 1.744** |
| **Herbaceous** | **5.357** | **0.021** | **-0.534 ± 0.239** |
| Soil temperature | 0.729 | 0.393 | 0.206 ± 0.241 |
| Soil moisture | 2.819 | 0.093 | 0.424 ± 0.253 |
| Soil temperature x Soil moisture | 0.677 | 0.411 | -0.181 ± 0.216 |
| Spatial filter no 1 | 3.805 | 0.051 | 5.678 ± 2.959 |
| Spatial filter no 27 | 1.317 | 0.251 | 2.956 ± 2.597 |
| **Females P/A** | **9.132** | **0.003** | **0.698 ± 0.233** |
| Yearlings P/A | 0.322 | 0.571 | 0.170 ± 0.297 |
|  |  |  |  |
| ***c. Yearlings*** |  |  |  |
| Intercept | 53.501 | <.0001 | -1.859 ± 0.303 |
| Bare soil | 3.060 | 0.080 | 0.361 ± 0.201 |
| Herbaceous | 0.001 | 0.981 | -0.007 ± 0.281 |
| Soil temperature | 0.038 | 0.845 | 0.059 ± 0.300 |
| Soil moisture | 0.705 | 0.401 | 0.235 ± 0.276 |
| Soil temperature x Soil moisture | 0.125 | 0.724 | -0.088 ± 0.244 |
| Spatial filter no 31 | 1.050 | 0.306 | 3.210 ± 3.175 |

Table S3. Continued

| *Variables* | χ^2^ *_1,155_* | *P* | β ± SE |
| --- | --- | --- | --- |
|  |  |  |  |
| Spatial filter no 32 | 3.571 | 0.059 | 6.498 ± 3.556 |
| Females P/A | 3.449 | 0.063 | 0.506 ± 0.269 |
| Males P/A | 1.198 | 0.274 | 0.329 ± 0.296 |
|  |  |  |  |

Table S4. GLM results for the socio-environmental-spatial presence/absence models for adult females, adult males, and yearlings in Formigal. Test statistics, derived from a likelihood-ratio test, and the standardized regression coefficient (*β* ± standard error), derived from the GLM are shown. Significant parameters are denoted in bold.

| *Variables* | χ^2^ *_1,230_* | *P* | β ± SE |
| --- | --- | --- | --- |
|  |  |  |  |
| ***a. Females*** |  |  |  |
| Intercept | 11.591 | 0.001 | -1.691 ± 0.559 |
| Bare soil | 1.352 | 0.245 | 1.126 ± 1.510 |
| Herbaceous | 2.935 | 0.087 | 1.537 ± 1.789 |
| Soil temperature | 0.070 | 0.792 | 0.083 ± 0.315 |
| Soil moisture | 0.108 | 0.743 | 0.109 ± 0.331 |
| Soil temperature x Soil moisture | 0.407 | 0.524 | -0.159 ± 0.249 |
| Spatial filter no 3 | 1.431 | 0.232 | -5.114 ± 4.345 |
| Spatial filter no 6 | 0.227 | 0.634 | 1.524 ± 3.206 |
| Spatial filter no 8 | 0.146 | 0.702 | -1.245 ± 3.262 |
| Spatial filter no 10 | 0.190 | 0.663 | -1.480 ± 3.411 |
| Males P/A | 1.665 | 0.197 | 0.331 ± 0.247 |
| Yearlings P/A | 0.634 | 0.426 | 0.372 ± 0.444 |
|  |  |  |  |
| ***b. Males*** |  |  |  |
| Intercept | 8.612 | 0.003 | -1.547 ± 0.609 |
| Bare soil | 0.008 | 0.930 | -0.033 ± 0.374 |
| Herbaceous | 0.009 | 0.926 | 0.028 ± 0.311 |
| Soil temperature | 0.025 | 0.874 | 0.049 ± 0.305 |
| **Soil moisture** | **4.201** | **0.040** | **0.559 ± 0.290** |
| Soil temperature x Soil moisture | 1.703 | 0.192 | 0.342 ± 0.267 |
| **Spatial filter no 1** | **11.503** | **0.001** | **-11.253 ± 3.521** |
| Spatial filter no 10 | 0.519 | 0.471 | -2.427 ± 3.376 |
| Spatial filter no 13 | 3.167 | 0.075 | -6.036 ± 3.472 |
| Spatial filter no 23 | 1.273 | 0.259 | -3.654 ± 3.260 |
| Females P/A | 1.156 | 0.282 | 0.291 ± 0.264 |
| Yearlings P/A | 0.016 | 0.900 | 0.073 ± 0.574 |
|  |  |  |  |

Table S4. Continued

| *Variables* | χ^2^ *_1,231_* | *P* | β ± SE |
| --- | --- | --- | --- |
|  |  |  |  |
| ***c. Yearlings*** |  |  |  |
| Intercept | 10.727 | 0.001 | -4.902 ± 1.497 |
| Bare soil | 0.760 | 0.383 | 4.010 ± 9.301 |
| Herbaceous | 1.192 | 0.275 | 5.209 ± 11.169 |
| Soil temperature | 2.217 | 0.137 | 0.895 ± 0.602 |
| Soil moisture | 1.517 | 0.218 | 0.648 ± 0.526 |
| Soil temperature x Soil moisture | 1.874 | 0.171 | -0.580 ± 0.424 |
| **Spatial filter no 2** | **4.621** | **0.032** | **-14.866 ± 6.915** |
| Spatial filter no 17 | 0.032 | 0.858 | 1.056 ± 5.895 |
| Spatial filter no 23 | 0.750 | 0.387 | 5.577 ± 6.440 |
| Females P/A | 0.310 | 0.577 | 0.256 ± 0.459 |
| Males P/A | 0.002 | 0.967 | -0.024 ± 0.590 |
|  |  |  |  |

Table S5. Spearman correlation coefficients between residuals of environmental models and spatial filters for the three lizard classes (adult females, adult males, yearlings) and a) Candanchú, b) Somport and c) Formigal. Significant filters with Moran’s *I* index > | 0.5 | are shown.

| *Variable* | *Moran’s I* | *Spearman ρ* | *P* |
| --- | --- | --- | --- |
|
|  |  |  |  |
| **a. Candanchú** |  |  |  |
| **Adult Females** |  |  |  |
| Spatial filter no 5 | 0.913 | 0.191 | 0.014 |
| Spatial filter no 8 | 0.867 | -0.174 | 0.025 |
| Spatial filter no 10 | 0.814 | -0.186 | 0.017 |
| Spatial filter no 13 | 0.719 | -0.170 | 0.029 |
| Spatial filter no 31 | -0.534 | -0.159 | 0.042 |
| **Adult Males** |  |  |  |
| Spatial filter no 2 | 0.996 | 0.223 | 0.004 |
| Spatial filter no 3 | 0.978 | -0.261 | 0.001 |
| Spatial filter no 5 | 0.913 | 0.263 | 0.001 |
| Spatial filter no 11 | 0.801 | 0.176 | 0.024 |
| Spatial filter no 12 | 0.755 | -0.192 | 0.014 |
| **Yearlings** | |  |  |
| Spatial filter no 3 | 0.978 | -0.226 | 0.004 |
| Spatial filter no 4 | 0.963 | 0.246 | 0.002 |
| Spatial filter no 9 | 0.834 | 0.158 | 0.042 |
|  |  |  |  |
|  |  |  |  |
| **b. Somport** |  |  |  |
| **Adult Females** |  |  |  |
| Spatial filter no 6 | 0.909 | 0.154 | 0.048 |
| Spatial filter no 27 | -0.539 | -0.165 | 0.034 |
| **Adult Males** |  |  |  |
| Spatial filter no 1 | 1.022 | 0.168 | 0.031 |
| Spatial filter no 27 | -0.539 | 0.207 | 0.008 |
|  |  |  |  |
| **Yearlings** |  |  |  |
| Spatial filter no 31 | -0.534 | 0.175 | 0.024 |
| Spatial filter no 32 | -0.570 | 0.186 | 0.017 |
|  |  |  |  |

Table S5. Continued

| *Variable* | *Moran’s I* | *Spearman ρ* | *P* |
| --- | --- | --- | --- |
|
|  |  |  |  |
| **Yearlings** | |  |  |
| Spatial filter no 31 | -0.534 | 0.175 | 0.024 |
| Spatial filter no 32 | -0.570 | 0.186 | 0.017 |
|  |  |  |  |
|  |  |  |  |
| **c. Formigal** |  |  |  |
| **Adult Females** |  |  |  |
| Spatial filter no 3 | 0.984 | 0.189 | 0.003 |
| Spatial filter no 6 | 0.926 | 0.185 | 0.004 |
| Spatial filter no 8 | 0.876 | -0.130 | 0.044 |
| Spatial filter no 10 | 0.819 | -0.143 | 0.026 |
| **Adult Males** |  |  |  |
| Spatial filter no 1 | 1.050 | -0.233 | 0.000 |
| Spatial filter no 10 | 0.819 | -0.142 | 0.027 |
| Spatial filter no 13 | 0.789 | -0.219 | 0.001 |
| Spatial filter no 23 | 0.612 | -0.131 | 0.042 |
| **Yearlings** | |  |  |
| Spatial filter no 2 | 1.017 | -0.223 | 0.001 |
| Spatial filter no 17 | 0.617 | -0.156 | 0.015 |
| Spatial filter no 23 | 0.612 | 0.210 | 0.001 |
|  |  |  |  |

Figure S2: Variation partitioning into independent Socio-Environmental (*Socio-Env*), Spatial (*Sp*), and shared components, for each population (SOM, CAN and FOR) and the three lizard classes. Numbers indicate the proportion of explained deviance for independent and shared components and circle size is proportional to the explained deviance.
